# Labor intensity as an independent risk factor for frailty in older farmers: evidence from a multi-region survey in Guizhou, China

**DOI:** 10.3389/fpubh.2026.1822228

**Published:** 2026-05-22

**Authors:** Yao Yan, Lin Zhou, Shuwen Huang, Fang He, Yinying Zhao, Jing Gong, Binxu Yang, Anmei Li, Juan Yang, Ran Zhang, Yu Huang, Hongya Xia, Xiuquan Shi, Jing Zhou

**Affiliations:** 1 Department of Nursing, The Affiliated Hospital of Zunyi Medical University, Zunyi, Guizhou, China; 2 School of Nursing, Zunyi Medical University, Zunyi, Guizhou, China; 3 Department of Epidemiology and Health Statistics, School of Public Health, Zunyi Medical University, Zunyi, Guizhou, China

**Keywords:** cross-sectional studies, farmers, frailty, non-linear dose-response relationship, rural population

## Abstract

**Background:**

The global agricultural labor force has declined from 41% in 1995 to 26% in 2023, while farming populations in Asian newly industrialized countries are aging rapidly. China, as a representative case, faces challenges in agricultural labor force renewal, with older farmers serving as “last guardians” through high-intensity labor. The prevalence of frailty among rural Chinese adults aged 60 years and older is 23.31%, significantly higher than in urban areas. However, previous studies have treated physical activity as homogeneous, failing to distinguish occupational agricultural labor from leisure-time activity. This study examined the association between agricultural labor intensity and frailty among older farmers, identifies risk thresholds, and provides evidence for promoting healthy aging during agricultural transformation.

**Methods:**

We conducted a multiregion cross-sectional survey in Guizhou Province, China. A total of 1,443 older farmers aged 60–79 years were selected using multifactor stratified sampling. Frailty was assessed using the Fried Frailty Phenotype. Labor intensity was quantified as total daily energy expenditure (kcal/day), calculated as body weight (kg) × daily working hours (h) × metabolic equivalent of task (MET). Multivariable logistic regression analyses were performed with stepwise adjustment for demographic, lifestyle, and health-related confounders.

**Results:**

The prevalence of frailty among older farmers was 19.7%, with significant regional variation. Advanced age, low educational attainment, malnutrition, polypharmacy, widowhood, and multiple pain sites were independently associated with frailty. A non-linear association was observed between agricultural labor intensity and frailty. Daily labor energy expenditure exceeding 1,752 kcal/day was associated with significantly increased frailty risk (OR = 3.596; 95% CI: 2.386–5.420; *P* < 0.001). Unlike leisure-time physical activity, high-intensity agricultural labor was independently associated with frailty.

**Conclusions:**

High labor intensity represents a strong, independent risk factor for frailty among older farmers. These findings underscore the urgent need for targeted interventions and occupational health guidelines to protect this vulnerable population from excessive physical workloads and promote healthy aging in rural communities.

## 1 Background

Data from the International Labour Organization (ILO) demonstrate that the proportion of the global agricultural labor force declined substantially from 41% in 1995 to 26% in 2023 (1). This rapid transition from agriculture is reshaping population age structures and altering the relationship between demographic change and agricultural production. Newly industrialized countries (NICs) in Asia, experiencing rapid urbanization and expansion of nonagricultural employment, are witnessing pronounced aging of their farming populations (2–4).

As a representative NIC (5), China faces significant challenges in agricultural labor force generational renewal (6). Official data indicate that by 2020, the proportion of farmers younger than 40 years had decreased to 19% and continues to decline (7). Model projections suggest that by 2030, the number of farmers aged 60 years and older will reach 123 million, including approximately 58.57 million aged 65 years or older (8). Notably, despite institutional shifts toward larger-scale farming, Chinese agriculture remains fundamentally characterized by smallholder production. Older farmers continue to identify strongly with farming and serve as the “last guardians” of the agricultural labor force through a strategy characterized by high labor intensity, low willingness to exit farming, and engagement in multiple occupations (8). Under population aging, labor-intensive agriculture faces a fundamental dilemma: a mismatch between labor supply and industry demands, further compounded by mechanization bottlenecks (9).

This phenomenon has triggered cascading health challenges. The prevalence of frailty among rural Chinese adults aged 60 years and older is 23.31% (10), significantly higher than rates reported in major urban areas such as Beijing and Shanghai (11, 12). Because older farmers are exposed to long-term heavy physical workloads and often lack occupational health protection (13), agriculture faces a dual burden: increasing average farmer age and progressive decline in the health of the aging agricultural workforce (14).

However, previous studies have largely treated physical activity as a homogeneous exposure, without distinguishing leisure-time physical activity from occupational agricultural labor. Additionally, few studies have quantified a specific safety threshold for labor intensity in older farmers or explored potential non-linear associations between labor intensity and frailty. Clarifying the effect of labor intensity on frailty and the social mechanisms that may modify this relationship is essential for promoting healthy aging and addressing the key scientific challenge of balancing productivity and sustainability during agricultural transformation. To address these gaps, we proposed three hypotheses:

Hypothesis 1: frailty prevalence differs across regions and socioeconomic levels among older farmers. Hypothesis 2: multiple individual characteristics, including advanced age, low education, malnutrition, polypharmacy, widowhood, and multiple pain sites, are independently associated with frailty. Hypothesis 3: a non-linear association exists between agricultural labor intensity and frailty risk, with higher labor intensity associated with increased frailty risk.

To test these hypotheses, we conducted a cross-sectional study among older farmers. By distinguishing occupational agricultural labor from general physical activity and providing the first quantitative safety threshold for labor intensity, this study seeks to fill these gaps and offer actionable evidence for targeted interventions.

## 2 Methods

### 2.1 Study design

This cross-sectional study was conducted in Guizhou Province, southwestern China, where more than 92% of the land is mountainous and the rural agricultural labor force constitutes a substantial population proportion. A cross-sectional design was selected to efficiently examine current frailty prevalence and its association with agricultural labor intensity among older farmers.

### 2.2 Study subjects and sampling methods

The sample size was calculated using the formula: n = $\frac{{\left(Z_{\alpha /2}\sqrt{2\bar{P}\left(1-\bar{P}\right)}+Z_{\beta }\sqrt{P_{0}\left(1-P_{0}\right)+P_{1}\left(1-P_{1}\right)}\right)}^{2}}{{\left(P_{1}-P_{0}\right)}^{2}}$,

According to previous literature, the prevalence of frailty among rural older farmers was reported to be 36.76%. The minimum required sample size was 993 participants. After accounting for an anticipated 20% invalid response and loss to follow-up rate, the target sample size was set at 1,192. A total of 1,443 eligible participants were finally enrolled.

This study used a multifactor stratified sampling design in Guizhou Province. Counties were stratified by geographic region (eastern, southern, western, northern) and relative economic level with reference to the World Bank's regional economic classification framework. Based on the 2023 provincial average GDP (54,200 yuan) as the benchmark, counties were classified into four tiers: developed (≥150%), relatively developed (100%−150%), moderately developed (50%−100%), and less developed (< 50%). Four representative cities were selected: Guiyang (south, developed), Zunyi (north, relatively developed), Tongren (east, moderately developed), and Bijie (west, less developed). This strategy accounted for geographic and socioeconomic heterogeneity and reduced sampling bias.

In China, the concept of “retirement” is ambiguous, and many older farmers remain engaged in agricultural work beyond the official retirement age. According to gerontological standards, individuals aged ≥60 years are defined as older adults. However, those aged ≥80 years (the oldest-old) typically experience severe physical decline and high care dependency, which may confound the association between agricultural labor and frailty (15, 16). We therefore restricted participants to 60–79 years to ensure they were physically capable of sustained farming while minimizing heterogeneity in physical function.

Inclusion criteria were: (1) aged 60–79 years; (2) clear consciousness, good comprehension and communication, and independent mobility to complete the investigation; (3) long-term engagement in agricultural work with no history of other occupations; (4) acknowledged and agreed to participate in this survey.

Exclusion criteria were: (1) severe cardiac, cerebrovascular, renal, pulmonary or other major organ diseases; (2) severe mental disorders; (3) inability to complete the questionnaire or dropout during investigation.

**Figure 1:**
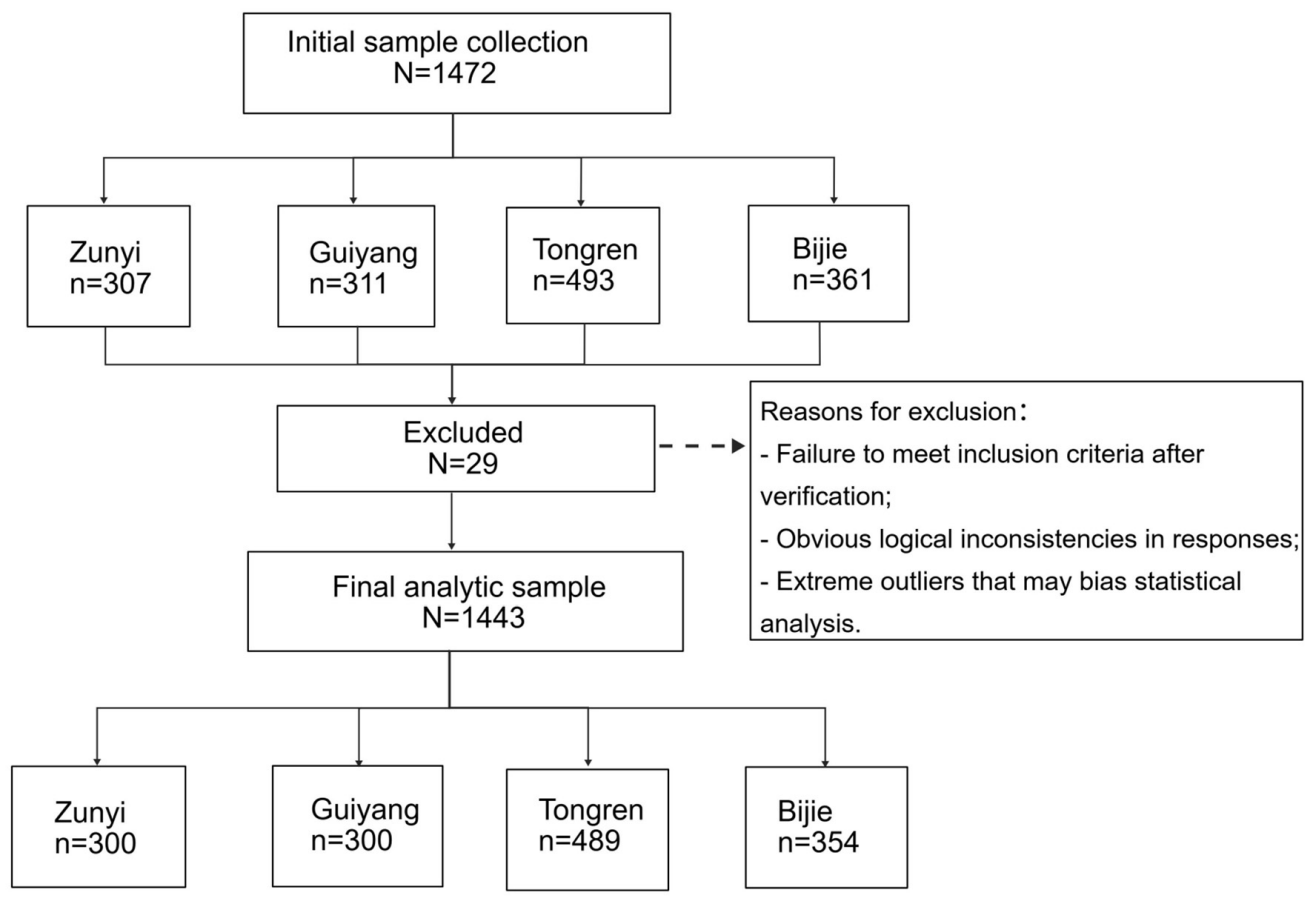
Flowchart of participant enrollment and selection.

Data were collected from March 1 to May 31, 2025, with a total of 1,443 valid participants included. The participant flowchart is shown in Figure 1.

### 2.3 Questionnaires

#### 2.3.1 Assessment of frailty

Frailty was assessed using the Fried Frailty Phenotype (FP) (17). The phenotype includes five criteria: slow walking speed, reduced grip strength, low physical activity, fatigue, and unintentional weight loss. Participants meeting three or more criteria were classified as frail, those meeting one or two criteria as prefrail, and those meeting none as non-frail. The FP evaluates physical frailty using objective and quantitative indicators and has strong predictive validity. The Cronbach's α of the scale in this study was 0.705.

#### 2.3.2 Labor intensity assessment and calculation

Labor intensity reflects actual physical workload and is commonly quantified by energy expenditure (kcal) or physiological load. In this study, daily labor intensity was quantified using the following energy expenditure formula derived from the Compendium of Physical Activities: Energy expenditure (kcal/day) = body weight (kg) × daily working hours (h) × metabolic equivalent of task (MET) (18). MET values were obtained from the Older Adult Compendium of Physical Activities, which provides energy costs specifically for adults aged 60 years and older (19). Based on this database, fixed MET values were assigned to common local agricultural activities: light activities (e.g., planting, potting, transplanting, and watering) were assigned 3.3 METs; moderate activities (e.g., digging, tilling, weeding, and plowing) were assigned 4.8 METs; and vigorous activities (e.g., heavy digging, composting, and carrying heavy loads) were assigned 7.3 METs (19).

During the field survey, a structured questionnaire collected detailed information on agricultural work types performed during the previous 6 months (each activity session lasting at least 30 min) and average daily working hours. For participants engaged in multiple activities of different intensities, the dominant intensity type was defined as the activity accounting for the largest proportion of working time. The corresponding MET value for that activity was then entered into the formula to calculate a continuous variable representing daily labor energy expenditure for subsequent analyses.

For example, consider a participant weighing 60 kg who worked an average of 5 h per day, including 1 h of planting/transplanting (3.3 METs), 3 h of weeding/tilling (4.8 METs), and 1 h of carrying heavy loads or vigorous digging (7.3 METs). According to our method, weeding/tilling (4.8 METs) was identified as the dominant activity because it accounted for the largest share of working time. The daily labor energy expenditure was calculated as: 60 kg × 5 h × 4.8 MET = 1,440 kcal/day. Thus, the estimated daily labor energy expenditure for this participant was 1,440 kcal/day.

#### 2.3.3 Geriatric depression scale (GDS-5)

The GDS-5, derived from the GDS-15, consists of the five items with the strongest predictive value for clinical depression (20). Responses are recorded as binary options (“yes” or “no”). Item 1 is reverse scored (yes = 0, no = 1), whereas items 2–5 are scored in the standard direction (yes = 1, no = 0). Total scores range from 0 to 5, with scores ≥2 indicating depressive symptoms; higher scores indicate greater symptom severity. The GDS-5 demonstrated a sensitivity of 0.94 and a specificity of 0.81 upon validation.

#### 2.3.4 Mini nutritional assessment-short form (MNA-SF)

The MNA-SF is widely used for early malnutrition screening due to its high sensitivity, specificity, and ease of administration (21). It consists of six items, including weight loss, acute stress, mobility, neuropsychological problems, appetite/digestion, and BMI, with a total score of 14. Scores of 0–7 indicate malnutrition, 8–11 indicate risk of malnutrition, and 12–14 indicate normal nutritional status. The instrument has demonstrated a sensitivity of 96%, specificity of 98%, and predictive value of 97%. The Cronbach's α of the scale in this study was 0.711.

#### 2.3.5 Demographic characteristics

Demographic and health-related characteristics included sex, age, marital status, living arrangement, educational level, body mass index (BMI), sleep duration, smoking status, alcohol consumption, number of chronic diseases, number of medications, number of pain sites, nutritional status, and depressive status.

### 2.4 Data collection

Data collection was conducted from March 1 to May 31, 2025. Investigators conducted one-on-one face-to-face interviews and entered responses directly into the Wenjuanxing online platform. All scales were used in their original validated form. Before formal investigation, all investigators received standardized training, and a pilot study was performed to ensure applicability. During interviews, standardized procedures were followed, with family members providing assistance only when necessary. Each participant received a small gift in appreciation of their participation. A total of 1,472 questionnaires were collected, of which 29 were excluded due to incomplete or inconsistent information, resulting in 1,443 valid responses and an effective response rate of 98%.

### 2.5 Quality control and survey implementation

Data collection was conducted across four cities in Guizhou Province—Guiyang, Zunyi, Tongren, and Bijie—with the involvement of 13 well-trained investigators, including eight postgraduate nursing students, one chief nurse specialist, two associate chief nurses, and two nursing supervisors. To ensure data quality, rigorous quality control procedures were implemented throughout the study. Validated instruments with established reliability and validity were selected, all investigators received standardized training, and the protocol was refined through a pilot survey. During fieldwork, one-on-one interviews and on-site verification were used. Potential participants were recruited through village committee announcements and household visits, and their status as farmers was confirmed through self-reported long-term agricultural engagement and verification by local village cadres. Two researchers independently entered the data, blinded to each other. Discrepancies were resolved by checking the original questionnaires. Logical verification was also performed. The participant flowchart (Figure 1) summarizes enrollment, screening, and final inclusion.

### 2.6 Statistical analysis

Participant characteristics are presented as frequencies and percentages for categorical variables, mean ± standard deviation (SD) for normally distributed continuous variables, and median [interquartile range (IQR)] for non-normally distributed continuous variables. Group differences in categorical variables were assessed using the chi-square test. Independent-samples *t* tests were used for normally distributed continuous variables, and the Mann–Whitney *U* test was used for non-normally distributed continuous variables.

Univariate analyses and multivariable binary logistic regression were performed to identify factors associated with frailty and evaluate the association between labor intensity and frailty risk. Three hierarchical models were constructed: Model 1 was unadjusted; Model 2 was adjusted for sociodemographic characteristics; and Model 3 was further adjusted for sociodemographic factors, lifestyle variables, and comorbidities. Restricted cubic spline (RCS) analysis was performed to explore potential non-linear relationships between continuous labor intensity and frailty risk. All statistical analyses were conducted using SPSS version 29.0 (IBM Corp., Armonk, NY, USA). A two-sided *P* value < 0.05 was considered statistically significant.

## 3 Results

### 3.1 Socio-demographic characteristics and univariate analysis of frailty prevalence

Among 1,443 older farmers, 19.7% were identified as frail, with notable regional variation across the four study sites. As shown in Table 1, frail participants differed significantly from non-frail participants across multiple sociodemographic and health-related characteristics (*P* < 0.05).

**Table 1:** Socio-demographic characteristics and univariate analysis associated with frailty prevalence.

Variable	Category	Non-frailty (*n* = 351)	Pre-frailty (*n* = 808)	Frailty (*n* = 284)	*t*/χ^2^/*Z*	*P*
Sex	Male	170 (48.4)	373 (46.2)	128 (45.1)	0.797	0.671
	Female	181 (51.6)	435 (53.8)	156 (54.9)		
Age(years)	–	64 (62, 70)	65 (62, 71)	69 (62, 74)	31.218	< 0.001
Educational level	Illiterate	164 (46.7)	455 (56.3)	193 (68.0)	43.183	< 0.001
	Primary school graduate	148 (42.2)	298 (36.9)	89 (31.3)		
	Junior high school and above	39 (11.1)	55 (6.8)	2 (0.7)		
Marital status	Married	314 (89.5)	711 (88.0)	224 (78.9)	31.824	< 0.001
	Divorced	27 (7.7)	82 (10.1)	37 (13.0)		
	Widowed	10 (2.8)	15 (1.9)	23 (8.1)		
Living arrangement	Living with spouse and children	109 (31.1)	245 (30.3)	93 (32.7)	7.268	0.297
	Living with spouse only	149 (42.5)	360 (44.6)	119 (41.9)		
	Living with children only	32 (9.1)	69 (8.5)	36 (12.7)		
	Living alone	61 (17.4)	134 (16.6)	36 (12.7)		
Monthly household income (RMB)	< 1,000	75 (21.4)	123 (15.2)	68 (23.9)	31.468	< 0.001
	1,000–1,999	221 (63.0)	603 (74.6)	190 (66.9)		
	2,000–2,999	28 (8.0)	27 (3.3)	7 (2.5)		
	≥3,000	27 (7.7)	55 (6.8)	19 (6.7)		
BMI	–	24.5 (22.2, 26.5)	24.2 (21.9, 26.6)	23.8 (21.2, 26.2)	6.652	0.032
Smoking status	Current smoker	296 (84.3)	639 (79.1)	234 (82.4)	7.148	0.128
	Current quitter	23 (6.6)	61 (8.3)	26 (9.2)		
	Never smoker	32 (9.1)	102 (12.6)	24 (8.5)		
Alcohol consumption status	Current drinker	234 (66.7)	459 (56.8)	186 (65.5)	21.090	< 0.001
	Current abstainer	10 (2.8)	64 (6.9)	23 (8.1)		
	Lifetime abstainer	107 (30.5)	285 (35.3)	75 (26.4)		
Number of comorbidities	–	1 (0, 1)	1 (0, 1)	1 (0, 1)	10.826	0.004
Number of regular medications	None	207 (59.0)	413 (51.1)	101 (35.6)	38.139	< 0.001
	Less than 3 types	123 (35.0)	316 (39.1)	143 (50.4)		
	3 or more types	21 (6.0)	79 (9.8)	40 (14.1)		
Number of body pain sites	–	0 (0, 1)	1 (0, 1)	1 (0, 2)	22.153	< 0.001
Depressive symptoms	No	318 (90.6)	713 (88.2)	230 (81.0)	14.378	< 0.001
	Yes	33 (9.4)	95 (11.8)	54 (19.0)		
Nutritional status	Malnutrition	87 (24.8)	231 (28.6)	135 (47.5)	44.422	< 0.001
	healthy	264 (75.2)	577 (71.4)	149 (52.5)		
Sleep duration	< 7 h	203 (57.8)	450 (55.7)	196 (69.0)	22.416	< 0.001
	7–9 h	122 (34.8)	324 (40.1)	78 (27.5)		
	≥9h	26 (7.4)	34 (4.2)	10 (3.5)		
Labor intensity (Kcal)		1,267 (613, 1964)	1,198 (594, 2112)	1,387 (789, 2,302)	12.203	0.002

Due to low cell counts (*n* < 10), the original “malnourished” category was combined with the “at risk of malnutrition” category into a single group labeled “Malnourished/at risk.”

### 3.2 Robustness analysis of the association between labor intensity and frailty

In fully adjusted multivariable logistic regression, labor intensity remained significantly associated with frailty both as a continuous variable (OR = 1.000, 95% CI: 1.000–1.001, *P* < 0.001) and as a categorical variable. Using the lowest intensity tertile (Q1) as reference, the highest tertile (Q3) was associated with substantially greater odds of frailty (OR = 3.596, 95% CI: 2.386–5.420, *P* < 0.001), supporting a clear dose–response relationship. Results were consistent across sequentially adjusted models (Table 2).

**Table 2:** Association between labor intensity and frailty across different models.

Variables	Model 1		Model 2		Model 3	
	OR (95% CI)	*P*	OR (95% CI)	*P*	OR (95% CI)	*P*
Labor intensity	1.188 (1.049–1.345)	0.006	1.440 (1.247–1.663)	< 0.001	1.000 (1.000–1.001)	< 0.001
**Labor intensity grouping (with Q1 as the reference group)**						
Q2	1.508 (1.083–2.100)	0.015	1.834 (1.286–2.615)	< 0.001	2.122 (1.456–3.093)	< 0.001
Q3	1.646(1.186–2.285)	0.003	2.473 (1.707–3.583)	< 0.001	3.596 (2.386–5.420)	< 0.001

Model 1: Crude. Model 2: Adjust: Sex, age, Education status, Marital status, Living arrangement, Monthly household income (RMB). Model 3: Adjust: Gender, age, Education status, Marital status, Living arrangement, Monthly household income (RMB), BMI, Smoking status, Alcohol status, Number of regular medications, Depressive symptoms, Nutritional status, Number of comorbidities, Number of body pain sites, Sleep duration.

### 3.3 Non-linear association between labor intensity and frailty: restricted cubic spline (RCS) analysis

**Figure 2:**
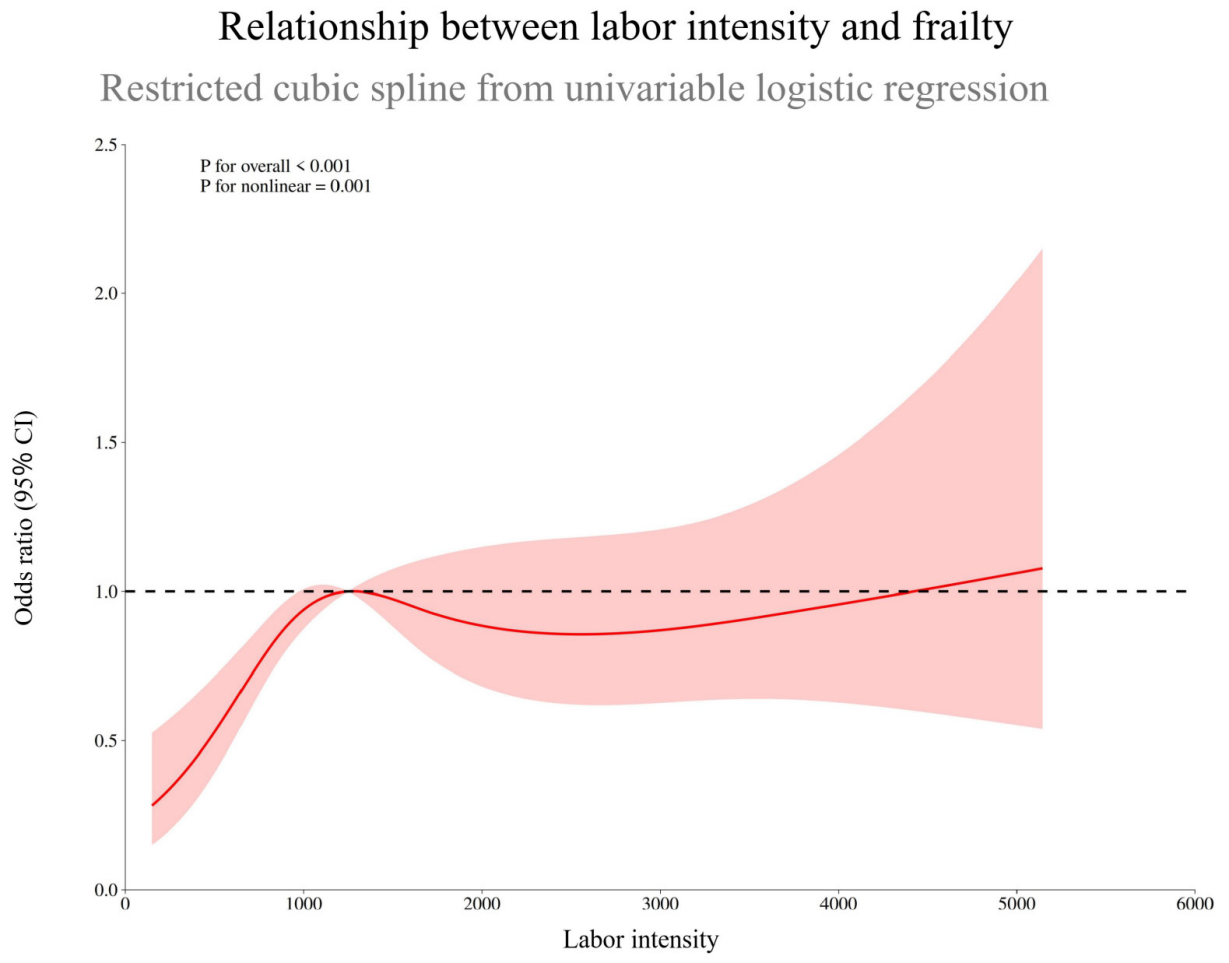
Non-linear association between labor intensity and frailty risk in older farmers.

Due to violation of the proportional odds assumption, frailty status was dichotomized as frail versus non-frail (including prefrail) for all subsequent modeling. Restricted cubic spline analysis revealed a significant J-shaped non-linear association between labor intensity and frailty (*P* for overall association < 0.001; *P* for non-linearity = 0.001). Frailty risk declined at low-to-moderate labor intensity but increased markedly at higher levels, as illustrated in Figure 2.

### 3.4 Multivariable logistic regression analysis of factors associated with frailty in older farmers

**Figure 3:**
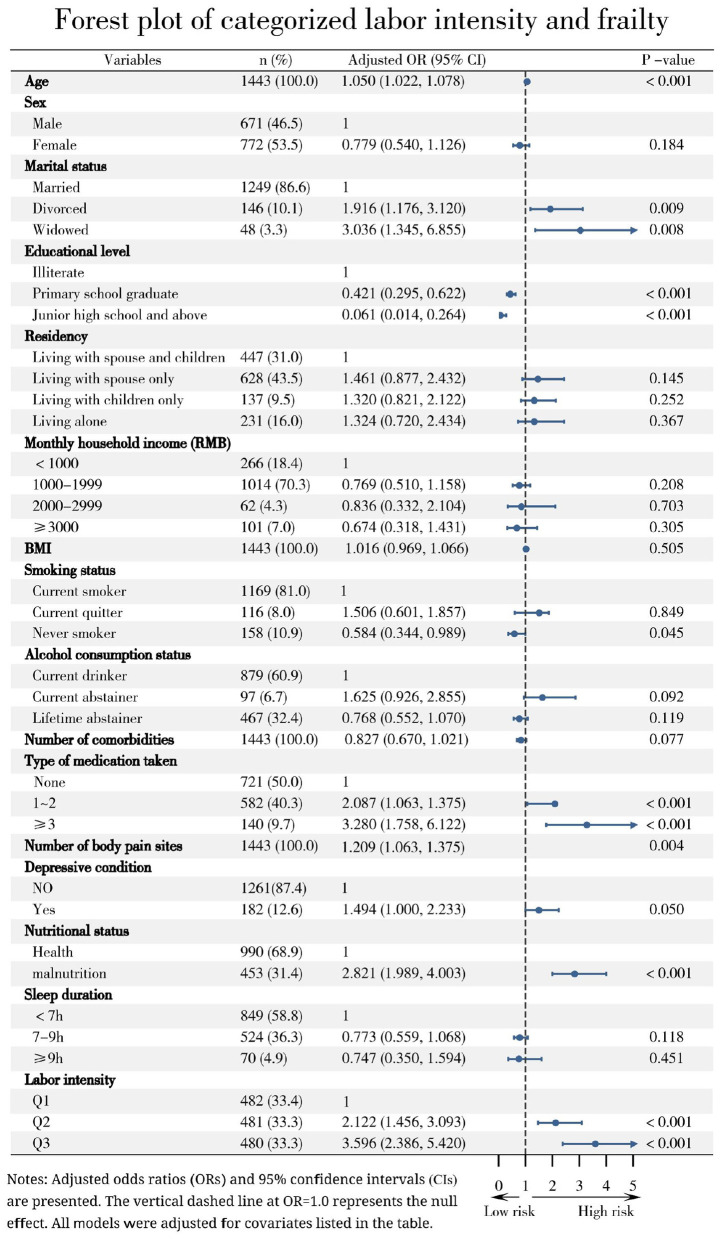
Association between categorized labor intensity and frailty in older farmers.

**Figure 4:**
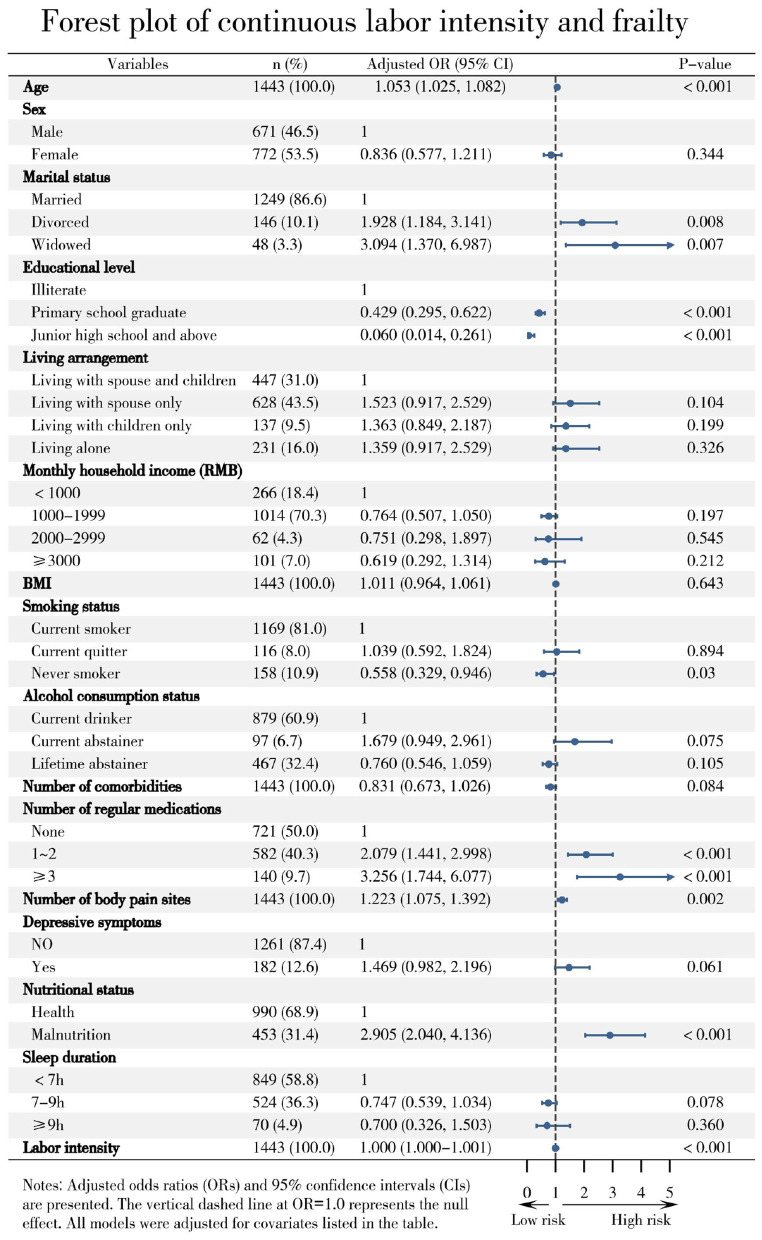
Association between continuous labor intensity and frailty in older farmers.

After full adjustment, higher labor intensity remained independently associated with increased odds of frailty in both continuous and categorical models. Other significant independent risk factors included older age, widowhood or divorce, polypharmacy, malnutrition, depressive symptoms, and multiple pain sites. Model fit was satisfactory (Hosmer–Lemeshow test: χ^2^ = 12.321, df = 8, *P* = 0.137) and no severe multicollinearity was detected (all VIF < 5) (Figures 3, 4.).

### 3.5 Effect modification and subgroup analyses

No significant interaction between age and labor intensity was observed (*P* for interaction = 0.407), indicating a stable association across age groups. In stratified analyses, the positive association between high labor intensity and frailty persisted in all subgroups. The association was stronger among women (OR = 5.23, 95% CI: 2.89–9.48) than men (OR = 2.70, 95% CI: 1.49–4.91), and varied by region, with the strongest effect observed in Zunyi (OR = 7.10, 95% CI: 2.38–21.16). Full subgroup estimates are presented in Table 3.

**Table 3:** Stratified analyses of the association between labor intensity and frailty.

Subgroup	Labor intensity	OR (95% CI)	*P*
By sex			
Male	Q2	2.076 (1.135–3.796)	0.018
Male	Q3	2.703 (1.490–4.906)	0.001
Female	Q2	2.357 (1.422–3.906)	0.001
Female	Q3	5.230 (2.885–9.481)	< 0.001
By nutritional status			
Well-nourished	Q2	1.868(1.089–3.205)	0.023
Well-nourished	Q3	3.880 (2.247–6.699)	< 0.001
Malnourished/at risk	Q2	2.160 (1.256–3.714)	0.005
Malnourished/at risk	Q3	2.864 (1.492–5.500)	0.002
By region			
Zunyi	Q2	3.510 (1.196–10.300)	0.022
Zunyi	Q3	7.101 (2.382–21.164)	< 0.001
Guiyang	Q2	3.083 (1.245–7.635)	0.015
Guiyang	Q3	3.367 (1.230–9.219)	0.018
Tongren	Q2	1.367 (0.745–2.509)	0.312
Tongren	Q3	3.135 (1.599–6.147)	0.001
Bijie	Q2	2.395 (1.061–5.410)	0.036
Bijie	Q3	4.773 (1.841–12.374)	0.001

## 4 Discussion

This study examined the prevalence and associated factors of frailty among older farmers, with a focus on labor intensity. We observed a 19.7% overall frailty prevalence, significant regional differences, and multiple individual factors independently linked to frailty. A non-linear relationship was identified between labor intensity and frailty, with a clear threshold at 1,752 kcal per day. These findings advance understanding of frailty in older farmers and provide evidence to support targeted health and occupational interventions for this population.

### 4.1 . Regional economic level was associated with frailty prevalence

The observed regional variations in frailty prevalence (16.3% in Guiyang to 22.9% in Bijie) are associated with profound socioeconomic disparities across Guizhou Province. Our findings align with previous research demonstrating that lower regional economic development is related to higher frailty burden among older farmers (22, 23). This geographic gradient emerges from multiple systemic factors, which are associated with frailty risk: inadequate healthcare infrastructure, limited access to preventive services, and unequal distribution of social resources in less developed regions (24). Economically advanced areas like Guiyang benefit from better medical facilities, higher health literacy rates, and more comprehensive social support systems, all of which correlate with reduced frailty risk (25). Conversely, regions such as Bijie face compounded challenges including population outmigration, insufficient public health investment, and persistent reliance on labor-intensive farming practices. The concentration of frailty in economically disadvantaged areas underscores the need for targeted interventions that address structural inequities rather than focusing solely on individual risk factors. These regional disparities highlight how agricultural modernization and economic development are associated with health outcomes in aging rural populations, suggesting that frailty prevention strategies must be tailored to local economic contexts and resource availability. Future policies should prioritize equitable resource allocation to bridge these geographic health gaps.

### 4.2 Individual factors independently associated with frailty

Our multivariable analysis identified several factors associated with frailty among older farmers, including advanced age, lower educational attainment, malnutrition, polypharmacy, widowhood, and multiple pain sites. These factors are linked to interconnected pathways that correlate with functional decline. Advanced age represents an immutable biological risk factor (26), while educational attainment serves as a powerful social determinant related to health literacy and access to resources (27). Nutritional challenges, which are common among those engaged in prolonged agricultural labor without adequate replenishment, may be part of a vicious cycle of energy depletion and functional impairment (28). Polypharmacy reflects both the burden of multiple chronic conditions and potential inappropriate medication use among older farmers, with medication side effects that may be associated with functional decline (29). Widowhood represents a significant psychosocial risk factor, as loss of spousal support is associated with reduced practical assistance and emotional well-being, which may correlate with frailty progression (30). Multiple pain sites further compound these challenges by limiting mobility and reducing participation in beneficial activities, both of which are linked to higher frailty risk (31). These findings emphasize the need for comprehensive geriatric assessment approaches that address multiple risk domains simultaneously rather than focusing on isolated factors. Future interventions should prioritize integrated care models that combine nutritional support, medication optimization, pain management, and social engagement strategies.

### 4.3 High-intensity agricultural labor is independently associated with increased frailty risk

Our study reveals a critical distinction between occupational agricultural labor and leisure-time physical activity regarding their relationship with frailty. While recreational exercise is associated with protective effects against functional decline (32–34), high-intensity farm work is related to unique risks through integrated biological, psychological, and social pathways. Biologically, prolonged repetitive movements, sustained fixed postures, and work on uneven terrain are associated with cumulative musculoskeletal strain that may exceed repair capacity over time (35, 36). Environmental stressors such as extreme temperatures are linked to these physiological challenges, and are associated with greater heat stress risk and impaired recovery processes (37). A lack of adequate rest periods is related to reduced tissue repair and physiological restoration, which are correlated with age-related functional decline (38). Psychologically, economic pressures and farm management responsibilities are associated with chronic stress, which is related to dysregulated neuroendocrine function and higher inflammatory markers, factors that are associated with frailty development (31). Socially, limited healthcare access, insufficient recovery time, and inadequate social support are linked to a negative feedback loop associated with functional impairment. This biopsychosocial framework is consistent with the observed higher frailty risk related to high-intensity agricultural labor. In contrast, leisure physical activity is usually voluntary, intermittent, health-oriented, and performed in a controlled environment with adequate recovery, thus being associated with protective effects on physical function. The key distinction lies in the involuntary nature, excessive duration, environmental stressors, and insufficient recovery periods characteristic of occupational farm work—factors absent in controlled recreational exercise settings. These findings are not aligned with conventional physical activity recommendations for older farmers and highlight the need for occupation-specific guidelines that account for contextual differences in physical exertion.

### 4.4 Targeted interventions and implementation strategies

To mitigate excessive workloads while maintaining agricultural productivity, we recommend the following targeted interventions. First, reduce physical workload through ergonomic optimization, using fruit and vegetable harvesting as an example. Field observations show that adopting conveyor belts and fixed-arm tilted boxes can cut forward bending by over 50% compared to manual carrying, effectively relieving lumbar spinal load (39). Since daily work beyond 8 h increases spinal compression and musculoskeletal risk, we recommend capping harvest time at 8 h per day. Simultaneously, promoting pot-based cultivation and taller crop varieties can ease spinal compression and avoid frequent deep bending. For tasks requiring kneeling or squatting, implement a work-rest cycle of 20 min of work followed by 10 min of walking. These ergonomic strategies, derived from harvesting scenarios, apply to other agricultural production types. Second, implement mandatory rest breaks during peak agricultural seasons: 30 min of rest after every 1.5 h of continuous work prevents cumulative fatigue and reduces injury risk (40). Women generally have lower physical capacity than men, a difference that becomes more pronounced with age. They also manage additional household responsibilities and tend to use gentler, more precise methods in farm work. Therefore, women and older farmers are better suited for low-intensity tasks like harvesting and watering, while younger men are more appropriate for high-intensity labor such as digging, plowing, and heavy lifting.

Third, strengthen nutritional support. During busy farming seasons, provide portable energy bars and encourage small, frequent, balanced meals to alleviate physical fatigue and counteract excessive energy consumption. Fourth, integrate regular health screening into village clinic services (41), including annual frailty assessment using the FRAIL scale and comprehensive pain evaluation for location, intensity, and duration—focusing on lower back, knees, and shoulders. For patients with moderate to severe pain, village doctors should provide education on proper body mechanics, offer topical or oral analgesics after safety screening, and schedule follow-ups every 3 months. Quarterly medication reviews are also recommended for older farmers taking three or more chronic medications to simplify complex regimens. These hierarchical interventions require collaboration among rural healthcare institutions, agricultural technical departments, and grassroots social organizations. Relevant policies should encourage agricultural mechanization in mountainous rural areas and establish phased retirement mechanisms that allow older farmers to gradually reduce farming intensity rather than quitting agricultural work abruptly.

### 4.5 Research and public health implications

Older farmers represent a population with limited attention in gerontological research, and the unique occupational context of agricultural labor has rarely been integrated into healthy aging frameworks. Our findings, while consistent with prior studies on the association between socioeconomic factors and frailty, extend existing understanding by highlighting the contextual differences between occupational and leisure physical activity—an aspect often overlooked in general physical activity guidelines. The divergent associations of occupational farm labor and leisure exercise with frailty risk emphasize that health recommendations for older farmers must be context-aware. Generalized physical activity guidelines, which primarily focus on the benefits of voluntary exercise, fail to account for the involuntary, high-intensity nature of agricultural labor, potentially leading to inappropriate health advice for this population. This gap in current guidelines underscores the need for occupation-specific health strategies that address the unique physiological and environmental challenges of agricultural work. From a public health perspective, our findings highlight the importance of addressing systemic factors that are associated with frailty risk among older farmers. Socioeconomic disparities, limited healthcare access, and the demanding nature of agricultural labor are interconnected, and interventions targeting only individual-level factors are unlikely to be effective. Instead, a holistic approach—integrating policy, healthcare, and occupational support—is needed to promote healthy aging in this vulnerable population.

### 4.6 Limitations

This study has several limitations that should be considered when interpreting the findings. First, its cross-sectional design prevents definitive causal inference about the temporal relationship between labor intensity and frailty. Although multiple potential confounders were adjusted for in the analysis, longitudinal research is necessary to clarify temporal order and further explore underlying mechanisms. Second, the healthy worker effect may have led to an underestimation of frailty prevalence. Because the sample included only currently active farmers aged 60–79 years, those who had already withdrawn from farming due to frailty, disability, or severe illness were excluded, potentially introducing selection bias, especially in regions with limited healthcare access. Third, despite the use of a validated calculation approach, labor intensity was based on self-reported information, which may be subject to recall bias and social desirability bias. Finally, the study was conducted in mountainous areas of Guizhou Province, which may limit the generalizability of the results to other agricultural regions with different topography, cropping systems, and levels of mechanization. Future prospective cohort studies, objective workload monitoring using wearable devices, and multi-regional comparative research are warranted to address these limitations and improve the external validity of the findings.

## 5 Conclusion

High labor intensity represents a strong, independent risk factor for frailty among older farmers. These findings underscore the urgent need for targeted interventions and occupational health guidelines to protect this vulnerable population from excessive physical workloads and promote healthy aging in rural communities. Based on our quantitative safety threshold (1,752 kcal/day), we propose the following actionable interventions: (a) reduce physical workload through ergonomic optimization and limit daily harvest time to 8 h; (b) enforce mandatory rest breaks with adequate hydration and energy supplementation; (c) assign lower-intensity tasks to women, older adults, and those with multiple pain sites or polypharmacy; and (d) strengthen nutritional support with portable energy bars and small, frequent meals during busy farming seasons; (e) integrate regular health screening into village clinic services, including annual frailty assessment, pain evaluation, quarterly medication reviews, and health education.

## Data Availability

The raw data supporting the conclusions of this article will be made available by the authors, without undue reservation.
